# A Scoping Review of Associations Between Cannabis Use and Anxiety in Adolescents and Young Adults

**DOI:** 10.1007/s10578-021-01280-w

**Published:** 2021-11-01

**Authors:** Colleen Stiles-Shields, Joseph Archer, Jim Zhang, Amanda Burnside, Janel Draxler, Lauren M. Potthoff, Karen M. Reyes, Faith Summersett Williams, Jennifer Westrick, Niranjan S. Karnik

**Affiliations:** 1 Department of Psychiatry and Behavioral Sciences, Rush University Medical Center, 1645 W. Jackson Blvd., Suite 302, Chicago, IL 60612 USA; 2 School of Medicine and Public Health, University of Wisconsin, Madison, WI USA; 3 Rosalind Franklin University of Medicine and Science, Chicago, IL USA; 4 Ann & Robert H. Lurie Children’s Hospital of Chicago, Chicago, IL USA

**Keywords:** Cannabis, Anxiety, Adolescents, Young adults, Review

## Abstract

Cannabis and anxiety are both rising issues that impact young people. This review seeks to explore the association between anxiety and cannabis in adolescents and young adults (AYA). A database search was run retrospectively from July 2020 through calendar year 2013. Articles had to present outcomes examining cannabis use and symptoms of anxiety, be written in English, contain samples with ≥ 50% who are age 25 or younger, and be published in a peer-reviewed journal. Forty-seven studies were identified that examined the relationship between anxiety and cannabis use. Twenty-three studies found a positive association that greater anxiety among AYA was associated with greater cannabis use. In contrast, seven studies found a negative association that greater anxiety was related to less cannabis use. And finally, 17 studies found no clear association between anxiety and cannabis use. Further research is needed to better understand the relationship between anxiety and cannabis use.

**Supplementary Information:**

The online version contains supplementary material available at 10.1007/s10578-021-01280-w.

Adolescence and young adulthood is a critical period of neurological and psychological development [1–3]. Due to the plasticity of the developing brain, young people are particularly susceptible to the environmental, social, and physiological factors that may contribute to the development and progression of mental illness and behavioral disorders [4]. According to the National Comorbidity Survey, the mean age of onset for any mental health disorder is 14 [5]. Mental and behavioral problems in adolescence may impair neurological and emotional development, and, if unaddressed, these issues will likely extend into adulthood [5, 6]. This study examined the current literature on two of the most prevalent mental and behavioral issues among adolescents and young adults (AYA): anxiety and cannabis use.

Anxiety disorders (e.g. generalized anxiety disorder, social anxiety disorder) are highly prevalent among AYA and are associated with adverse outcomes later in life. The estimated prevalence of anxiety disorders in the United States is 31.9% for adolescents (ages 13–18) [7] and 14.7% for young adults (ages 18–25) [8]. The median age of onset for any anxiety disorder worldwide is 17, making early recognition and prevention crucial [9]. Some anxiety disorders begin at even younger ages due to changes in social relationships in childhood and adolescence [10, 11]; the median age of onset for separation and social anxiety disorders are 8 and 13, respectively [9]. Furthermore, anxiety disorders put AYA at greater risk for major depression, illicit substance dependence, reduced self-esteem, and educational underachievement in adulthood [11–13]. In addition to individual sequelae, anxiety disorders pose significant societal burdens, including lost work productivity and high medical resource use [14]. Indeed, the mean total annual cost per patient for pediatric anxiety is $6405, with costs increasing with elevated symptoms of anxiety [15].

Cannabis use is also pervasive among AYA, particularly so with increased legalization in the United States, and has likewise been under scrutiny from researchers due to potential long-term consequences. According to the 2019 Monitoring the Future national survey, 35.7% of 12th grade students, 28.8% of 10th grade students, and 11.8% of 8th grade students reported marijuana use within the past year [16]. Furthermore, according to the 2014 National Survey on Drug Use and Health (NSDUH), 1.09% of 12–17 year-olds reported daily cannabis use, as did 6.51% of 18–25 year-olds, the highest rate of any age category [17]. In 2014, only four states and the District of Columbia (D.C.) had legalized recreational cannabis use [18], but now, as of April 2021, recreational use has been legalized in 17 states, D.C., and Guam [19]. As cannabis legalization has expanded since the most recent national cannabis use rates were reported, it is possible that rates are even higher today [20, 21]. Furthermore, while there is conflicting evidence on the sequelae of early cannabis use [22], some studies have suggested a link between early use and adverse outcomes in adulthood such as illicit drug use, suicide attempts, and lower educational attainment [23, 24].

Both anxiety disorders and cannabis use have complex etiologies involving psychosocial, physiological, and environmental factors contributing to their development and progression [25–27]. It has been posited that anxiety may contribute to the etiology of cannabis use, or visa versa. However, this hypothesis is controversial, and the significance and directionality of the relationship has not been fully established [28–30]. Some research has found that cannabis may temporarily reduce anxiety symptoms, but the long-term effects of frequent cannabis use on mental health are unclear [29, 31, 32]. Cannabis use is common among those with anxiety disorders, but it is yet to be determined whether cannabis use itself contributes to the development and progression of anxiety disorders or if anxiety symptoms lead to cannabis use and potential dependence [31, 33]. Yet, despite this ambiguity, pediatricians today often receive questions from AYA, parents and caregivers about the potential use of cannabis as a treatment option for AYA patients with anxiety and other mental health problems [34].

The purpose of this scoping review was to describe the current state of scientific literature examining the relationship between anxiety and cannabis use among adolescents and young adults. The population of interest for this review includes AYA up to 25 years of age due to the high demand for information on cannabis as a treatment option for anxiety in pediatric clinics, which often provide care for patients well into young adulthood [35]. This topic is of particular importance to pediatricians due to the reported consequences of both early cannabis use and early onset of anxiety [11–13, 23, 24]. A literature search of recently published articles was conducted to elucidate current knowledge, identify gaps in the literature, and provide directions for future research. It is our intention that the information gathered in this review will inform clinicians and researchers interested in understanding and furthering current knowledge on cannabis use and anxiety in AYA.

## Methods

### Search Strategy

This review was conducted and reported following the Preferred Reporting Items for Systematic Reviews and Meta-Analyses (PRISMA) extension for scoping reviews [36] and was registered prior to data extraction in Open Science Framework (https://osf.io/zhxem/). A comprehensive literature search was run in the following databases: PubMed/MEDLINE, Scopus, CINAHL, PsycINFO and Google Scholar. Both controlled vocabulary (i.e., Mesh terms) and keywords were searched. No restrictions were placed on the search in terms of language, date of publication, or geography (to best reflect the variable legal status of cannabis across different states). Animal studies were excluded. Additionally, a hand search was conducted of the reference lists of selected included studies and similar review articles. The search strategy was conducted collaboratively by two authors (CSS, JZ) and a trained medical librarian (JW) and the literature search was conducted by a trained medical librarian (JW) in July 2020. A reproducible search strategy is listed in Appendix 1.

### Inclusion and Exclusion Criteria

For inclusion in the review, articles had to: (1) present outcomes directly examining real-life cannabis use and symptoms of anxiety (e.g., studies that broadly examined internalizing symptoms [combining depression and anxiety]; (2) be written in English; (3) contain samples with ≥ 50% who are 25 years of age or under; and (4) be published in a peer-reviewed journal. As this review aimed to examine associations in real-world experiences, studies with methodologies that induced cannabidonal experiences in a lab environment were excluded. Technical validation papers, conference abstracts, review papers, and studies with subjects primarily older than 25 years of age were also excluded. At the full text review stage, articles published prior to 2013 were also excluded to target the recent literature associating cannabis use with anxiety symptoms in AYA.

### Study Selection

Covidence, an online systematic review service partnered with Cochrane [37], was utilized to facilitate study selection. From the literature search results, two reviewers independently screened all titles and abstracts against the inclusion criteria. Next, two independent reviewers reviewed full-text articles. Inclusion discrepancies at both stages were resolved through consensus with a third reviewer.

### Data Extraction

Reviewer teams (JA, JZ, AB, JD, LMP, KMR, FSW) extracted data independently and in duplicate from all eligible studies using an online extraction form designed by the lead author (CSS) and housed on Google Forms. Discrepancies were resolved through review by the lead author.

### Data Synthesis

A systematic narrative framework was utilized, classifying results based upon their findings associating anxiety with cannabis use (i.e., “Positive Association,” “Negative Association,” and “Unclear/No Association”). To contextualize the findings and promote future inclusive research methodological and reporting practices, study and sample characteristics and primary outcomes were included.

## Results

### Included Studies

**Fig. 1:**
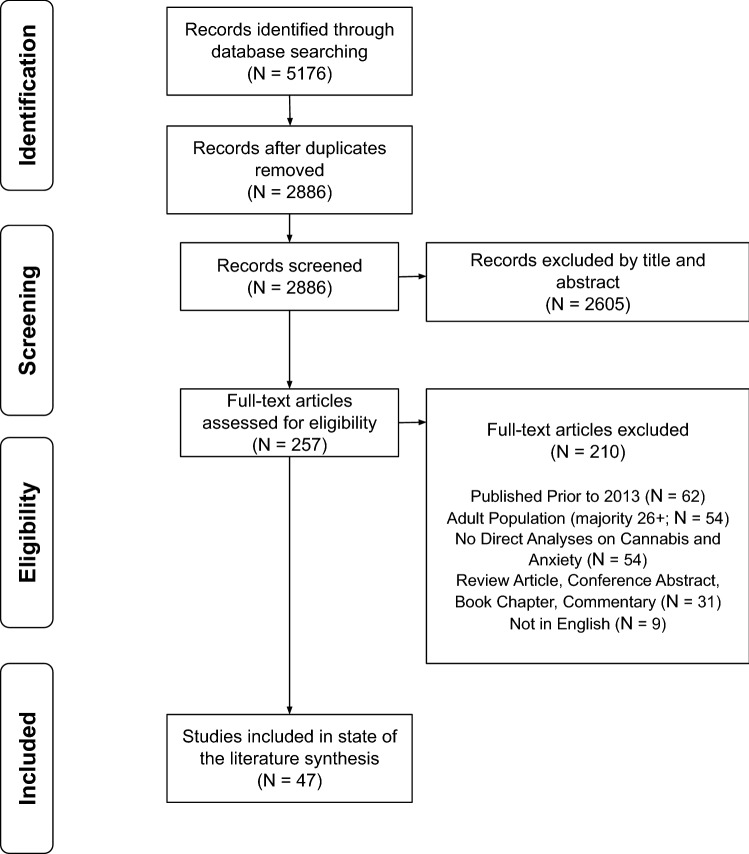
PRISMA flow chart

See Fig. 1 for the PRISMA flow diagram. Following the removal of duplicate articles, 2886 titles and abstracts were independently reviewed in duplicate. Two hundred fifty-seven full-text articles were reviewed in duplicate for inclusion, with 47 articles selected for data extraction. Of the 47 studies: 23 reported a positive association (e.g., higher anxiety associated with earlier onset of use, higher frequency of use, and/or greater cannabis-associated problems); seven reported a negative association (e.g., higher anxiety associated with less use); and 17 reported an unclear or no association between anxiety and cannabis use. See Tables 1, 2 and 3 for the study characteristics.

**Table 1:** Positive associations between anxiety and cannabis use frequency, onset, and/or problems

First Author, Year	Country	Study setting	Age, *M (range)*	Sample size, *N (%female)*	Race	Ethnicity	Cannabis measurement	Anxiety measurement	Association
Cerdá et al., 2013	United States	High School	6.7 at baseline (1st grade-age 19)	503 (0%)	56% Black; 41% White; 3% Asian, Mexican, Mixed Race	Partially combined with Race Reporting/NR	Self-Report: SRA; 16-item Substance Use Scale based on the National Youth Survey	Self-Report: CBCL	Recent, cumulative anxiety associated with earlier cannabis initiation (compared to control)
Cloak et al., 2015	United States	Community Environment	18.3–19.4 Across Groups (13–23)	122 (38.6–49%)	NR	NR	Clinical Interview: Structured Interview Biometric Data: Urine Toxicology Screen	Self-Report: SCL-90R; BPRS Biometric data: Cortisol Levels	Longer duration of abstinence associated with less Anxiety; Higher lifetime cannabis use associated with more Anxiety symptoms
Degenhardt et al., 2013	Australia	High School	14.9 at baseline (14.9–29.0)	1756 (53%)	NR	NR	Self-Reported Use	Clinical Interview: CIS-R	Daily, weekly, and occasional cannabis users at higher likelihood of meeting criteria for anxiety
Duperrouzel et al., 2018	United States	Urban/City Environment, High School	15.41 (14–17)	250 (43.6%)	89.6% Hispanic; 4.8% Black; 4.4% White; 1.2% Other	Combined with Race Reporting	Self-Report: Drug-Use History Questionnaire	Self-Report: DASS-21	Initial cannabis use frequency positively associated with subsequent changes in anxiety
Ecker et al., 2014	United States	University/College	19.68 (18–23)	230 (63.0%)	85.7% White; 5.7% African American; 4.3% Mixed; 3.0% Asian American; 1.3% Other	92.6% Non-Hispanic/Latino; 7.6% Hispanic/Latino	Self-Report: MUF	Self-Report: SIAS	General, Social anxiety positively correlated with cannabis-related problems; General anxiety associated with cannabis use frequency
Ecker et al., 2018	United States	University/College	20.32 (18-NR)	244 (76.2%)	74.2% non-Hispanic Caucasian; 13.5% African American/Black; 5.7% Asian; 5.0% Multiracial 1.2% Hispanic Caucasian; .8% American Indian	Combined with Race Reporting	Self-Report: TFB; MPS	Self-Report: SIAS	Social anxiety associated with problem severity
Foster et al., 2016	United States	Community Environment	21.01 (18–36)	148 (36.5%)	59.7% Caucasian; 24.8% African American; 8.1% Mixed; 3.4% Asian; 3.4% Other; .67% Native American	NR	Self-Report: Marijuana Use Questionnaire; MPS	Self-Report: SIAS; BSI	General, Social anxiety associated with cannabis problems
Hellemans et al., 2019	Canada	University/College	20 (NR)	1043 (62.5%)	57.4% White; 9.7% Black; 9.4% Arab/West Asian; 8.6% Asian; 4.9% South Asian; 4.2% Other; 2.3% Indigenous; 2.0% South East Asian; 1.4% Latin American/Hispanic	Combined with Race Reporting	Self-Report: CUDIT-R	Self-Report: BAI	Problematic cannabis use associated with higher anxiety scores, but moderated by gender (stronger for females)
Hill et al., 2017	United States	Urban/City Environment, Rural Environment	Longitudinal (9–30)	1229 (49.9%)	89% White; 7.25% African American; 3.75% American Indian	NR	Self-Report: YAPA	Self-Report: CAPA (< 16); YAPA (19 +)	Anxiety disorders during childhood/early-adolescence (ages 9–16) and late-adolescence (19–21) were more prevalent amongst persistent, problematic users
Hines et al., 2020	United Kingdom	Community Environment	24 (NR)	1087 (53.4%)	5.3% “Black or minority ethnic group”	Combined with Race Reporting	Self-Reported Use	Clinical Interview: CIS-R	Use of high-potency cannabis associated with a moderate elevation in likelihood of generalized anxiety disorder (p = .02)
Kaasbøll et al., 2018	Norway	High School	NR (13–17)	36714 (49.3%)	NR	NR	Self-Reported Use	Self-Report: HSCL	Cannabis users endorsed higher anxiety symptoms
Keith et al., 2015	United States	University/College	19.9 (NR)	1776 (56.5%)	47.3% White; 29.6% Asian/Pacific Islander; 11.0% Hispanic; 8.5% Multiracial; 6.2% Black	Combined with Race Reporting	Self-Reported Use	Self-Reported Diagnostic and Treatment history	Anxiety associated with marijuana use
Keough et al., 2018	Canada	University/College	20.18 (NR)	91 (55%)	NR	44% Other; 42% Canadian/European; 14% South Asian	Self-Report: MPS	Self-Report: AS	Anxiety sensitivity positively correlated with cannabis-related problems
Laguerre et al., 2015	France	High School	17.54 (NR)	336 (63.1%)	NR	NR	Self-Reported Use	Self-Report: ASA-27	Separation anxiety higher in cannabis users than nonusers
Leadbetter et al., 2019	Canada	Community Environment	Baseline: 15.52 (12–18; Longitudinal)	662 (52%)	85% White	NR	Self-Reported Use; Clinical Interview: MINI	Clinical Interview: BCFPI	Cannabis use disorder associated with higher anxiety symptoms at ages 26–27 only
Otten et al., 2016	The Netherlands	Community Environment	13.02, 15.73, 18.54 (longitudinal)	1424 (53.1%)	NR	NR	Self-Reported Use	Self-Report: Anxiety Problems Scale of ASEBA	For short allele carriers (not non-carriers), use positively associated with higher and increasing levels of anxiety
Pang et al., 2017	United States	High School	14.67, 15.12, 15.51,16.14 (9th-10th Grade)	2057 (58%)	Across Substance Types: 48.3–56.7% Hispanic/Latino; 14.8–17.7% White; 6.85–8.7% Asian; 2.8–4.9% Black/African American; 3.8–5.2% Native Hawaiian or Pacific Islander; 4.4–7.3% Multiracial; 7.0–10.0% Other	Combined with Race Reporting	Self-Report: YRBSS; Monitoring the Future	Self-Report: CASI; RCADS	Anxiety sensitivity symptoms associated with more negative cannabis effects of cannabis; Higher GAD symptoms initially report more positive cannabis effects, but have a slower increase in these effects across time
Rusby et al., 2019	United States	High School	14.4 (8th -10th grade)	466 (52.8%)	44% White, Non-Hispanic; 38% Hispanic; 18% Other or multiple races	Combined with Race Reporting	Self-Report: OHT	Self-Reported Mood (EMA)	Recent cannabis use associated with significantly greater anxious mood lability
Schuster et al., 2019	United States	Urban/City Environment	21.79 (18–25)	76 (44.7%)	63.2% White; 15.8% Black; 11.8% More than one race; 9.2% Other	NR	Self-Report: CUDIT-R; TFB; MEEQ Biometric Data: Urine Toxicology Screen	Self-Report: MASQ	Current anxious symptoms, arousal associated with greater cannabis use dependency in young adults who use cannabis at least weekly
Stapinski et al., 2016	Chile	Urban/City Environment, High School	14.5 (12–18)	2508 (44.5%)	NR	NR	Self-Reported Use	Self-Report: RCADS	Baseline generalized anxiety associated with cannabis use frequency at 18-month follow-up
Thompson et al., 2018	Canada	Urban/City Environment	15.5 at T1 (12–22; 18–29 ranges across timepoints)	662 (50.3%)	NR	NR	T1: Self-Reported Use T6: Clinical Interview: MINI	Self-Report: BCFPI	Chronic users reported higher anxiety symptoms; for young adults, chronic users reported more anxiety symptoms than all other classes except occasional users
Villarosa-Hurlocker et al., 2019	United States	University/College	20.24 (NR)	2034 (69.1%)	NR	67.95% White, Non-Hispanic; 15.88% Hispanic/Latino Ethnicity	Self-Report: B-MACQ	Self-Report: SIAS; BFNE	When controlling for all other predictors, social anxiety associated with more cannabis-related problems
Wolitzky-Taylor et al., 2016	Unites States	High School	14.1 (NR)	3002 (54.1%)	47.4% Hispanic; 6.7% Multiracial; 16.6% Asian; 16.1% Caucasian; 4.9% African American; 4.1% Native Hawaiian or Pacific Islander; 1% American Indian or Alaska Native	Combined with Race Reporting	Self-Report: CAST	Self-Report: RCADS	Negative urgency mediated associations between cannabis use and generalized anxiety disorder, panic disorder, social phobia

**Table 2:** Negative associations between anxiety and cannabis use

First Author, Year	Country	Study setting	Age, *M (range)*	Sample size, *N (% female)*	Race	Ethnicity	Cannabis measurement	Anxiety measurement	Association
Ali et al., 2016	France	High School, Vocational school	NR (14–20)	5069 (54%)	NR	NR	Self-Report: ESPAD	Self-Report: SURPS	Higher anxiety sensitivity associated with decreased current frequency of use
Bierhoff et al., 2019	United States	University/College	20.49 (18–25)	2397 (64.7%)	65.5% White; 21.8% Black; 6.9% Asian; 5.8% Other	92.2% non-Hispanic; 7.8% Hispanic	Self-Reported Use	Self-Report: SAS	Use associated with lower levels of anxiety symptoms
Di Blasi et al., 2015	Italy	High School	16.39 (14–19)	1305 (51.4%)	NR	NR	Self-Report: CUPIT	Self-Report: SIAS	Higher social anxiety associated with lower use
Schmitts et al., 2015	Belgium	High School	15.61 (14–18)	877 (50%)	NR	~ 85% Belgian; NR% Italian, Moroccan, Turkish, Other minorities	Self-Report: MUF	Self-Report: STAI-CH; LSAS-CA-SR	Higher levels of social anxiety at first time point were related to a reduced probability of cannabis initiation at next time point
Schmitts et al., 2015	Belgium	High School	16.64 (14–21)	130 (42.3%)	NR	NR	Self-Report: MUF; CPQ-A; MEEQ	Self-Report: STAI-CH; LSAS-CA-SR	Cannabis users endorsed less social anxiety
Schmitts et al., 2016	Belgium	High School	15.7 (14–18)	1343 (325 cannabis users; 45.6%)	NR	~ 85% Belgian; NR% Italian, Moroccan, Turkish, Other minorities	Self-Report: MUF; CPQ-A; MEEQ	Self-Report: LSAS-CA-SR	Higher social anxiety associated with expected negative behavioral effects of cannabis use, less cannabis use
Schmitts et al., 2018	Belgium	High School	15.54 (14–18)	611 (49.3%)	NR	~ 88% Belgian; NR% Italian, Moroccan, Turkish, Other minorities	Self-Report: MUF; CPQ-A; MEEQ	Self-Report: STAI-CH; LSAS-CA-SR	Higher social anxiety at first time point less likely to have used cannabis by third time point, and have a higher level of negative behavioral effect expectancies

**Table 3:** Unclear or no associations between anxiety and cannabis use

First Author, Year	Country	Study setting	Age, *M (range)*	Sample size, *N (% female)*	Race	Ethnicity	Cannabis measurement	Anxiety measurement	Association
Buckner et al., 2016	United States	University/College	20.2 (18–29)	276 (79.7%)	76.1% Non-Hispanic White; 12.0% Non-Hispanic African American; 4.7% Multiracial; 2.5% Asian or Asian American; 2.2% Hispanic White; 1.1% Other; 1.0% American Indian or Alaska Native; .4% Hispanic African American	Combined with Race Reporting	Self-Report: MUF; Investigator-developed questionnaire adapted from Gonzalez & Skewes	Self-Report: SIAS	Cannabis problems more likely related to solitary use than social anxiety experiences
Butler et al., 2019	Canada	High School	NR	6550 (51.7%)	NR	71.5% “Non-minority ethnicity”	Self-Reported Use	Self-Report: GAD-7	Anxiety associated with cannabis use frequency in one model, but not after including flourishing in later models
Cerdá et al., 2016	United States	High School	NR (13–19)	503 (0%)	56% Black; 41% White; 3% Asian, Mexican, Mixed Race	Partially combined with Race Reporting/NR	Self-Report: SRA; 16-item Substance Use Scale based on the National Youth Survey	Self-Report: CBCL	Higher anxiety and affective problems not associated with use
Cloutier et al., 2016	United States	Community Environment	16.2 (12–17)	56 (41.1%)	83.9% Caucasian; 5.4% African American; 7.1% multiracial; 3.6% “Other”	9.1% Hispanic/Latino	Self-Report: AADIS; TMMQ	Self-Report: RCADS (social anxiety subscale); Youth Self-Report – Anxiety problems subscale	No association with anxiety across measures of use frequency
Colder et al., 2019	United States	Community Environment	12 (11–12)	387 (55%)	83.1% non-Hispanic Caucasian; 9.1% African American	Partially combined with Race Reporting/NR	Self-Reported Use Self-Report: MMQ; MACQ	Self-Report: SIAS	Elevations in social anxiety were not associated with coping motives, or with cannabis use or problems
Ecker et al., 2014	United States	University/College	20.28 (18-NR)	158 (75.3%)	77.2% Caucasian; 8.9% African American; 8.2% Mixed; 5.1% Asian; .6% Other	8.2% Hispanic/Latino	Self-Report: Daily Drug-Taking Questionnaire; MPS	Self-Report: Social Phobia Scale	Non-significant correlation between cannabis use frequency and social anxiety
Elkington et al., 2016	United States	Urban/City Environment, Community Environment	12.58 (9–16)	340 (51.2%)	PHIV + : 57.8% Black; 30.1% Hispanic; 12.1% Other PHIV-: 49.3% Black; 32.1% Hispanic; 18.6% Other	Combined with Race Reporting	Clinical Interview: DISC-IV	Clinical Interview: DISC-IV	Frequency of use not associated with having an anxiety disorder at baseline, follow-up points
Gage et al., 2015	United Kingdom	Rural Environment	16, 18 (Prospective assessments)	4561 (NR)	NR	NR	Self-Reported Use	Clinical Interview: CIS-R	No significant relationship
Gillen et al., 2016	United States	Residential military-style program for youth who have dropped out of high school	16.74 (16–19)	185 (0%)	54.1% White; 24.3% Black; 18.4% Did not report; 1.6% Another ethnic group; 1.1% Hispanic; .5% Asian	Combined with Race Reporting	Self-Report: CRAFFT; MMQ	Self-Report: Personality Inventory for Youth (Fear and Worry Subscale)	No significant relationship
Grunberg et al., 2015	United States	University/College	18.30–18.38 (18–21)	375 (46.9%)	73% White; 18.7% Multi-Racial; 3.6% Asian; 3.3% Hispanic; .6% East Indian; .3% Black; .3% Middle Eastern .3% Pacific Islander	Combined with Race Reporting	Self-Report: TLFB	Self-Report: ASEBA	Initial cannabis use not correlated with anxiety at current or later time point
Khoddam et al., 2016	United States	High School	14.1 (NR)	3383 (53%)	45.9% Hispanic or Latino; 15.8% Asian; 15.3% White; 5.9% Multiracial; 5.6% Other; 4.9% Black/African American; 3.3% Native Hawaiian/Pacific Islander; 0.9% American Indian/Alaska Native	Combined with Race Reporting	Self-Reported Use	Self-Report: RCADS	Anxiety symptoms not associated with use over and above conduct problems
Ninnemann et al., 2017	United States	High School	16.09 (NR)	964 (56%)	31% African American; 29% White; 28% Hispanic; 12% Other	Combined with Race Reporting	Self-Report: MTF	Self-Report: Screen for Child Anxiety-Related Emotional Disorders	No significant relationship
Osuch et al., 2013	Canada	Urban/City Environment, Community Environment	19 (16–26)	429 (63.2%)	NR	NR	Self-Report: NIDA Modified ASSIST	Self-Report: STAI	No significant relationship
Phillips et al., 2018	United States	University/College	20.32 (18–25)	300 (60%)	69% Caucasian (Non-Latino/Hispanic); 15% Latino/Hispanic; 6% African American; 6% Multi-racial; 2% Asian; 2% Pacific Islander/Hawaiian	Combined with Race Reporting	Self-Report: Marijuana Use Measure; MPI Biometric Data: Urine Toxicology Screen	Self-Report: BAI; SIAS	No significant relationship
Rahm-Knigge et al., 2019	United States	University/College	18.92 (NR)	1005 (67.6%)	83.75 White; 5.1% Multi-racial; 4.0% Asian; 3.0% Do not wish to respond; 2.2% Black or African American; .9% American Indian or Alaskan Native; 0.9% Native Hawaiian or Other Pacific Islander	81% Not Hispanic or Latino; 15.6% Hispanic or Latino; 2.5% Do not wish to respond; .9% Missing	Self-Report: RBI	Self-Report: SAIS	No direct association, but certain profiles (Low Social Interaction Anxiety High Urgency) may be more likely to endorse use
Walters et al., 2018	United States	University/College	20.37 (18–25)	891 (69.6%)	69% White; 15% Black/African American; 8% Hispanic/Latino; 6% Other; 2% Asian/Pacific Islander	Combined with Race Reporting	Self-Report: CORE Alcohol and Drug Survey—Short form	Self-Report: PAI	No significant relationship
Wright et al., 2016	United States	University/College	21.2 (18–25)	84 (46.4%)	Control: 60% Caucasian; Cannabis Users: 67% Caucasian	NR	Self-Report: TLFB; Clinical Interview: Semi-structured interview	Self-Report: STAI	Overall, no association; Female cannabis users more likely to experience anxiety symptoms (p = .04)

### Positive Association

#### Study Characteristics

Twenty-three epidemiologic and population-based studies identified a positive association between higher anxiety levels with cannabis use frequency, onset, and/or problems. These studies were conducted in eight countries: United States of America (13; 56.5%), Canada (4; 17.4%), Australia (1; 4.3%), Chile (1; 4.3%), France (1; 4.3%), The Netherlands (1; 4.3%), Norway (1; 4.3%), and the United Kingdom (1; 4.3%). Notably, two studies from Canada derived from the Victoria Healthy Youth Survey, which prospectively assessed a community sample of 662 youth for ten years [38, 39]. Study settings included high schools, university or college campuses, community environments, urban/city environments, and rural environments. Cannabis use and/or problems were assessed via: validated self-report questionnaires (12; 52.2%), self-reported frequency of use (7; 30.4%), or a multi-method approach (clinical interview with biometric data [1; 4.3%] or with self-reported use [1; 4.3%]; validated self-report questionnaire with biometric data [1; 4.3%]; self-reported use at one time point and a clinical interview at a later time point [1; 4.3%]). For the assessment of anxiety, validated self-report questionnaires (17; 73.9%), clinical interviews (3; 13%) ecological momentary assessment (1; 4.3%), self-reported diagnostic and treatment history (1; 4.3%), and a combination of validated self-report questionnaire and biometric data (1; 4.3%) were utilized.

#### Sample Characteristics

Sample sizes ranged from 76 to 36,714, with females comprising 0–76.2% of the samples. No studies reported gender options beyond “male” and “female,” with one exception which noted that three participants identified their gender as “Other”; however, these participants were excluded from gender-based analyses [40]. While all studies had at least half of their samples composed of participants 25 years of age or under, the range of included ages spanned six to 36 years of age. Seven studies (30.4%) did not present any racial or ethnic identity information about their samples, with two more studies only providing the percentage of the sample that identified with one identity (e.g., “85% White”). Four samples (17.4%) had a minoritized racial or ethnic minority as the most represented group in their study.

#### Primary Outcomes

All 23 studies reported positive associations between anxiety or symptoms of anxiety with cannabis use, frequency of use, onset, and/or problems. Anxiety was associated with earlier cannabis initiation [41], higher cumulative lifetime use [42], use dependency [43], and general endorsement of use (as opposed to frequency of use) [44]. Further, frequent cannabis users were more likely than infrequent or non-users to meet criteria for anxiety disorders [45], endorse higher anxiety symptoms [39, 46], and/or display increases in anxiety over time [39, 47] or at a specific time period in adulthood (i.e., 26–27 years of age) [38]. Recent cannabis use was associated with greater anxious mood lability [48]. Similarly, longer abstinence from cannabis use was associated with less anxiety [42]. Also in line with these findings, those with anxiety disorders in childhood or adolescence were more likely to report persistent and problematic cannabis use as young adults (compared to those without problematic cannabis use who did not experience anxiety disorders early in life) [49].

Studies also reported associations with specific types of anxiety, as well as potential mediators and moderators. Generalized and social anxiety were both associated with cannabis-related problems (but not use) [50–52], with social anxiety also being associated with more problem severity [53] and generalized anxiety associated with use frequency (as opposed to endorsement of any use) [50, 54]. Cannabis users were also more likely to endorse higher separation anxiety in adolescence compared to non-users [55]. Anxiety sensitivity was associated with cannabis-related problems [56] and more negative effects of cannabis [57]. Use of higher potency cannabis was associated with moderate elevations in the likelihood of meeting criteria for generalized anxiety disorder [58]. Gender moderated the relationship between problematic use with anxiety (stronger for females) [40]. Certain genotypes (i.e., short allele carriers vs. non-carriers) were also identified as moderators of the relationship between higher cannabis use and anxiety symptoms [59]. Finally, negative urgency (the tendency to act impulsively in the face of stress) mediated associations between cannabis use with generalized anxiety disorder, panic disorder, and social phobia [60].

### Negative Association

#### Study Characteristics

Seven epidemiologic and population-based studies identified a negative association between anxiety levels and cannabis use. These studies were conducted in four countries: Belgium (4; 57.1%), France (1; 14.3%), Italy (1; 14.3%), and the United States of America (1; 14.3%). Notably, the studies occurring in Belgium were from the same research group and high school setting. Study settings included high schools, vocational schools, and university or college campuses. Cannabis use and/or problems were assessed via validated self-report questionnaires (6; 85.7%) or self-reported frequency of use (1; 14.3%). All seven studies used validated self-report questionnaires to assess anxiety.

#### Sample Characteristics

Sample sizes ranged from 130 to 5069, with females comprising 42.3–64.7% of the samples. No studies reported gender options beyond “male” and “female.” The samples were mostly adolescent, ranging from 14 to 19 years of age. Three studies (42.9%) did not present any racial or ethnic identity information about their samples, with three more studies only providing the percentages of the sample based on ethnicity (i.e., Hispanic/non-Hispanic; Belgian/Italian, Moroccan, Turkish, Other minorities).

#### Primary Outcomes

All seven studies reported a negative association between anxiety and cannabis use. Indeed, cannabis use in the last 30 days was associated with lower levels of overall anxiety symptoms compared to non-users [61] and lifetime cannabis use was associated with less social anxiety compared to non-users [62]. Specific types of anxiety were also noted with these negative associations. Namely, higher anxiety sensitivity was associated with decreased frequency of cannabis use [63]. Higher social anxiety was associated with non-use compared to moderate and risky cannabis use [64, 65] and was also associated with a reduced probability of cannabis initiation over time [66, 67]. Social anxiety was hypothesized as protective due to its association with more expectations of negative behavioral effects from the use of cannabis [65, 67].

### Unclear or No Association

#### Study Characteristics

Seventeen epidemiologic and population-based studies did not identify significant evidence to associate anxiety and cannabis use. These studies were conducted in three countries: the United States of America (14; 82.4%), Canada (2; 11.8%), and the United Kingdom (1; 5.9%). Study settings included high schools, university or college campuses, community environment, urban/city environment, rural environment, and a residential military-style program for youth who have dropped out of high school. Cannabis use and/or problems were assessed via: validated self-report questionnaires (10; 58.8%), self-reported frequency of use (3; 41.2%), clinical interview (1; 5.9%), or a multi-method approach (validated self-report questionnaire with self reported use [1; 5.9%], with biometric data [1; 5.9%], or with a clinical interview [1; 5.9%]). Fifteen studies (88.2%) used validated self-report questionnaires to assess anxiety, and two studies used a clinical interview (11.8%).

#### Sample Characteristics

Sample sizes ranged from 56 to 6,550, with females comprising 0–79.7% of the samples. One study did not report sex and no studies reported gender options beyond “male” and “female.” While all studies had at least half of their samples composed of participants 25 years of age or under, the range of included ages spanned 11 to 29 years of age. Two studies (11.8%) did not present any racial or ethnic identity information about their samples, with two studies (11.8%) only providing the percentage of the sample that identified with one identity (e.g., “60% Caucasian;” “71.5% Non-minority ethnicity”). Four samples (23.5%) had a minoritized racial or ethnic population as the most represented group in their study.

#### Primary Outcomes

None of the 17 studies identified significant associations between anxiety and cannabis use. Specifically, anxiety and social anxiety were not significantly associated with use [68–78], use frequency [79–81], problems [69, 74, 82], or coping motives [69, 83]. Unclear findings also occurred, such as an association appearing in one model of a study, but losing significance as additional variables were added to later models [84]. Other factors were also identified that potentially are associated both with having anxiety and/or cannabis use, such as conduct problems [78], solitary cannabis use [82], having certain personality profiles (e.g., low social interaction anxiety with high urgency) [75], or being a female cannabis user [77].

## Discussion

The current scoping review synthesized the recent literature examining potential associations between anxiety and cannabis use in AYA. The studies that met inclusion criteria were internationally representative, included longitudinal and cross-sectional data, and ranged in sample sizes from 56 to 36,714. High variability was present in terms of methodological approaches, including the assessment of anxiety and cannabis use to collecting and reporting sample characteristics (e.g., gender, race). Most crucially to this review, studies indicated mixed findings regarding the relationship between anxiety and cannabis use in AYA. Indeed, nearly half of the studies identified a positive association between higher anxiety levels with cannabis use frequency, onset, and/or problems; a little over three quarters were unable to identify a significant association; and a minority identified a negative association, such that those with higher anxiety (often social anxiety) had lower use and/or problems.

The clinical implications of the current state of research are mixed. Clinicians often meet with adolescents who use cannabis presenting with anxiety as the reason for their cannabis use. With the legalization of cannabis across multiple jurisdictions in the U.S., the potential for adolescents’ expanded access to cannabis is highly likely. Clinicians need to be vigilant in asking adolescents about cannabis and make attempts to understand the dynamics of its use. One helpful clinical question is to query the youth whether the anxiety began before or after the initiation of cannabis. This might help guide the discussion and clinical decisions about the intersection of these experiences. Finally, it is likely that if the adolescent tries to reduce cannabis use, they might experience transient rebound anxiety symptoms. Discussion of how to address these symptoms might help youth who wish to make a change in their patterns of use.

There is substantial need for additional research in this domain. In particular, there is a need to conduct a large, prospective cohort study that tracks the onset of anxiety as well as cannabis, and clearly delineates the connections between these phenomena. In addition, it is also important to examine differential predictors of both anxiety and substance use among marginalized or underrepresented groups of adolescents. The use of consistent methodological approaches, measures, and outcomes would increase generalizability of these studies and allow linkage in the event that studies of various populations are done independently.

There are several limitations to the current review. First, it was focused on anxiety and cannabis and, as such, excluded many other domains of psychiatric experience and substance use. The degree of comorbidity for both anxiety and cannabis use often intersects with other phenomena that may not be well represented in this scoping review. Second, this review focused on literature and research published in English. Both cannabis and anxiety are very much global experiences and there is a possibility that some important literature might have been excluded. Third, it should be noted that several studies used relatively limited sampling, heterogeneous definitions, and a range of sample sizes that in aggregate might reduce generalizability.

## Summary

Anxiety and cannabis use are highly common among adolescents and young adults (up to age 25). The rising movement to legalize recreational use of cannabis is likely to further expand the presence of this substance in various forms. In this scoping review of the literature, 47 studies were identified that examined the relationship between anxiety and cannabis use. Of these studies, a plurality of 23 studies found a positive association that greater anxiety among AYA was associated with greater cannabis use. In contrast, seven studies found a negative association that greater anxiety was related to less cannabis use. And finally, 17 studies found no clear association between anxiety and cannabis use. In aggregate, these findings present a mixed picture with unclear outcomes. There is a significant need to more rigorously examine the association between anxiety and cannabis use, and to pay particular attention to factors that might be unique to underrepresented groups.

### Supplementary Information

Below is the link to the electronic supplementary material.

Electronic supplementary material 1 (DOCX 38 kb)

## Appendix 1: Literature Search Strategy

Reproducible Search Strategy: PubMed

(((anxiety disorders[MeSH Terms]) OR (anxiety OR panic OR agoraphobia OR phobia))

AND ((((“Cannabinoids”[Mesh]) OR “Cannabis”[Mesh]) OR “Marijuana Abuse”[Mesh]) OR “Marijuana Smoking”[Mesh] OR “Medical Marijuana”[Mesh] OR CBD OR cannabis OR cannabinoid* OR cannabidiol* OR cesamet OR dronabinol OR endocannabinoid* OR hashish OR hash OR levonantradol OR marijuana OR marihuana OR Nabilone OR THC OR tetrahydrocannabinol OR pot OR weed))

AND (adolescent[MeSH Terms] OR Adolescen* OR “high school” OR “middle school” OR “elementary school” OR “secondary school” OR “secondary education” OR “grade school” OR juvenile* OR kid OR kids OR minor* OR paediatric* OR pediatric* OR pediatrics OR teen* OR young OR youth*))

NOT ((“animals”[MeSH Terms] NOT “humans”[MeSH Terms]) OR mice[tiab] OR rat[tiab] OR rats[tiab])

Search Terms

**Table:**

anxiety disorders[MeSH Terms] OR anxiety OR panic OR agoraphobia OR phobia	“Cannabinoids”[Mesh] OR “Cannabis”[Mesh] OR “Marijuana Abuse”[Mesh] OR “Marijuana Smoking”[Mesh] OR “Medical Marijuana”[Mesh] OR CBD OR cannabis OR cannabinoid* OR cannabidiol* OR cesamet OR dronabinol OR endocannabinoid* OR hashish OR hash OR levonantradol OR marijuana OR marihuana OR Nabilone OR THC OR tetrahydrocannabinol OR pot OR weed	adolescent[MeSH Terms] OR Adolescen* OR “high school” OR “middle school” OR “elementary school” OR “secondary school” OR “secondary education” OR “grade school” OR juvenile* OR kid OR kids OR minor* OR paediatric* OR pediatric* OR pediatrics OR teen* OR young OR youth*
NOT animals		

## Abbreviations

AYA: Adolescents and young adults
PRISMA: Preferred Reporting Items for Systematic Reviews and Meta-Analyses
