# Supplementary material for: A Scoping Review of Associations Between Cannabis Use and Anxiety in Adolescents and Young Adults
Source: Child Psychiatry Hum Dev. 2021 Nov 1;54(3):639–58. doi: 10.1007/s10578-021-01280-w (PMC9310430; doi:10.1007/s10578-021-01280-w)
Supplement: Supplementary file 1 — Electronic supplementary material 1 (DOCX 38 kb) [file 10578_2021_1280_MOESM1_ESM.docx]

| First Author, Year | Country | Study Setting | Age, *M (Range)* | Sample Size,  *N (% Female)* | Race | Ethnicity | Cannabis Measurement | Anxiety Measurement | Association |  |
| --- | --- | --- | --- | --- | --- | --- | --- | --- | --- | --- |
| **Higher Anxiety Associated with Lower Cannabis Use** | | | | | | | | | | |
| Ali et al., 2016 | France | High School, Vocational school | NR  (14-20) | 5,069 (54%) | NR | NR | Self-Report: ESPAD | Self-Report: SURPS | Higher anxiety sensitivity scores associated with decreased current frequency of use (p<.001) |  |
| Bierhoff et al., 2019 | United States | University/ College | 20.49 (18-25) | 2397 (64.7%) | 65.5% White; 21.8% Black; 6.9% Asian;  5.8% Other | 92.2% non-Hispanic; 7.8% Hispanic | Self-Reported Use | Self-Report: SAS | Use associated with lower levels of anxiety symptoms (p=.007) |  |
| Di Blasi et al., 2015 | Italy | High School | 16.39 (14-19) | 1305 (51.4%) | NR | NR | Self-Report: CUPIT | Self-Report: SIAS | Higher social anxiety associated with lower use (p<.01) |  |
| Schmits et al., 2015 | Belgium | High School | 15.61 (14-18) | 877 (50%) | NR | ~85% Belgian; NR% Italian, Moroccan, Turkish, Other minorities | Self-Report: MUF | Self-Report: STAI-CH; LSAS-CA-SR | Higher levels of social anxiety at T1 were related to a reduced probability of cannabis initiation at T2 (p = 0.03) |  |
| Schmitts et al., 2015 | Belgium | High School | 16.64 (14-21) | 130 (42.3%) | NR | NR | Self-Report: MUF; CPQ-A; MEEQ | Self-Report: STAI-CH; LSAS-CA-SR | Cannabis users endorsed less social anxiety (p = .01) |  |
| Schmitts et al., 2016 | Belgium | High School | 15.7 (14-18) | 1,343 (325 cannabis users; 45.6%) | NR | ~85% Belgian; NR% Italian, Moroccan, Turkish, Other minorities | Self-Report: MUF; CPQ-A; MEEQ | Self-Report: LSAS-CA-SR | The more social anxiety scores increased, the more teens expected negative behavioral effects of cannabis use (p = .005) and less likely to report cannabis use (p = .05) |  |
| Schmitts et al., 2018 | Belgium | High School | 15.54 (14-18) | 611 (49.3%) | NR | ~88% Belgian; NR% Italian, Moroccan, Turkish, Other minorities | Self-Report: MUF; CPQ-A; MEEQ | Self-Report: STAI-CH; LSAS-CA-SR | Teenagers with a higher level of social anxiety at T1 were less likely to have used cannabis at T3, partially due to their higher level of negative behavioral effect expectancies |  |
| **Higher Anxiety Associated with Higher, Earlier Cannabis Use and/or Problems** | | | | | | | | | | |
| Cerdá et al., 2013 | United States | High School | 6.7 at baseline (longitudinal design 1st grade-age 19) | 503 (0%) | 56% Black; 41% White; 3% Asian, Mexican, Mixed Race | Partially combined with Race Reporting/ NR | Self-Report: SRA; 16-item Substance Use Scale based on the National Youth Survey | Self-Report: CBCL | Recent and cumulative anxiety both associated with earlier cannabis initiation compared to control group |  |
| Cloak et al., 2015 | United States | Community Environment | 18.3-19.4 Across Use Groups (13-23) | 122 (38.6-49% Across Use Groups) | NR | NR | Clinical Interview: Structured Interview about Use History Biometric Data: Urine Toxicology Screen | Self-Report: SCL-90R; BPRS Biometric data: Cortisol Levels | Longer duration of abstinence associated with less Anxiety (p=0.017); More lifetime cannabis use associated with more Anxiety symptoms (p=0.021) |  |
| Degenhardt et al., 2013 | Australia | High School | 14.9 at baseline (Longitudinal design; 14-9-29.0) | 1756 (53%) | NR | NR | Self-Reported Use | Clinical Interview: CIS-R | Daily, weekly, and occasional cannabis users at higher likelihood of meeting criteria for anxiety |  |
| Duperrouzel et al., 2018 | United States | Urban/ City Environment, High School | 15.41 (14-17) | 250 (43.6%) | 89.6% Hispanic; 4.8% Black; 4.4% White; 1.2% Other | Combined with Race Reporting | Self-Report: Drug-Use History Questionnaire | Self-Report: DASS-21 | Positive association between initial levels of cannabis use and subsequent changes in anxiety (p = 0.024) |  |
| Ecker et al., 2014 | United States | University/ College | 19.68 (18-23) | 230 (63.0%) | 85.7% White; 5.7% African American; 4.3% Mixed; 3.0% Asian American; 1.3% Other | 92.6% Non-Hispanic/Latino; 7.6% Hispanic/Latino | Self-Report: MUF | Self-Report: SIAS | General and Social anxiety positively correlated with cannabis-related problems (ps<.01) but cannabis use frequency only associated with general anxiety (p<.01) |  |
| Ecker et al., 2018 | United States | University/ College | 20.32 (18-NR) | 244 (76.2%) | 74.2% non-Hispanic Caucasian; 13.5% African American/Black; 5.7% Asian; 5.0% Multiracial 1.2% Hispanic Caucasian; .8% American Indian | Combined with Race Reporting | Self-Report: Timeline Follow Back; Marijuana Problems Scale | Self-Report: Social Interaction Anxiety Scale (SIAS) | Social anxiety associated with problem severity, but not frequency of use |  |
| Foster et al., 2016 | United States | Community Environment | 21.01 (18-36) | 148 (36.5%) | 59.7% Caucasian; 24.8% African American; 8.1% Mixed; 3.4% Asian; 3.4% Other; .67% Native American | NR | Self-Report: Marijuana Use Questionnaire; Marijuana Problems Scale | Self-Report: SIAS; Brief Symptom Inventory | General and Social anxiety associated with cannabis problems (ps <.001), but not use |  |
| Hellemans et al., 2019 | Canada | University/ College | 20 (NR) | 1043 (62.5%) | 57.4% White; 9.7% Black; 9.4% Arab/West Asian;  8.6% Asian; 4.9% South Asian; 4.2% Other; 2.3% Indigenous; 2.0% South East Asian;  1.4% Latin American/Hispanic | Combined with Race Reporting | Self-Report: CUDIT-R | Self-Report: BAI | Family history of anxiety associated with earlier age of cannabis use and problematic use (ps = .001); Problematic cannabis use associated with higher anxiety scores, but moderated by gender (stronger for females, P < 0.001) |  |
| Hill et al., 2017 | United States | Urban/ City Environment, Rural Environment | Longitudinal (9-30) | 1229 (49.9%) | 89% White; 7.25% African American; 3.75% American Indian | NR | Self-Report: YAPA | Self-Report: Child and Adolescent Psychiatric Assessment (CAPA; <16); Young Adult Psychiatric Assessment (YAPA; 19+) | Anxiety disorders during childhood/early-adolescence (ages 9-16) and late-adolescence (19-21) were more prevalent amongst persistent, problematic users |  |
| Hines et al., 2020 | United Kingdom | Community Environment | 24 (NR) | 1087 (53.4%) | 5.3% "Black or minority ethnic group" | Combined with Race Reporting | Self-Reported Use | Clinical Interview: Clinical Interview Schedule–revised | Use of high-potency cannabis associated with a moderate elevation in likelihood of generalized anxiety disorder (p = .02) |  |
| Kaasbøll et al., 2018 | Norway | High School | NR (13-17) | 36,714 (49.3%) | NR | NR | Self-Reported Use | Self-Report: HSCL | Cannabis users endorsed higher anxiety symptoms (p < 0.001) |  |
| Keith et al., 2015 | United States | University/ College | 19.9 (NR) | 1776 (56.5%) | 47.3% White; 29.6% Asian/Pacific Islander; 11.0% Hispanic; 8.5% Multiracial; 6.2% Black | Combined with Race Reporting | Self-Reported Use | Self-Reported Diagnostic and Treatment history | Anxiety associated with marijuana use, but not frequency of use |  |
| Keough et al., 2018 | Canada | University/ College | 20.18 (NR) | 91 (55%) | NR | 44% Other ethnicity; 42% Canadian/European;  14% South Asian | Self-Report: Marijuana Problems Scale | Self-Report: Original 16-item Anxiety Sensitivity (AS) Index | Anxiety sensitivity positively correlated with cannabis-related problems (p < .01) |  |
| Laguerre et al., 2015 | France | High School | 17.54 (NR) | 336 (63.1%) | NR | NR | Self-Report: Frequency of Cannabis Use | Self-Report: Adult Separation Anxiety Questionnaire (ASA-27) | Separation anxiety higher in cannabis users than nonusers (p = .018) |  |
| Leadbetter et al., 2019 | Canada | Community Environment | Baseline: 15.52 (12-18; Longitudinal) | 662 (52%) | 85% White | NR | Self-Reported Use;  Clinical Interview: Mini‐International Neuropsychiatric Interview (MINI) | Clinical Interview: Brief Child and Family Phone Interview (BCFPI) | Cannabis use disorder associated with higher anxiety symptoms at ages 26–27 only |  |
| Otten et al., 2016 | The Netherlands | Community Environment | 13.02, 15.73, 18.54 (longitudinal) | 1,424 (53.1%) | NR | NR | Self-Reported Use | Self-Report: Anxiety Problems Scale of the Achenbach Youth Self-Report | For short allele carriers (not non-carriers), use positively associated with higher and increasing levels of anxiety |  |
| Pang et al., 2017 | United States | High School | 14.67, 15.12, 15.51,16.14 (9th-10th Grade) | 2,057 (58%) | Across Substance Types: 48.3-56.7% Hispanic/Latino; 14.8-17.7% White; 6.85-8.7% Asian; 2.8-4.9% Black/African American; 3.8-5.2% Native Hawaiian or Pacific Islander; 4.4-7.3% Multiracial; 7.0-10.0% Other | Combined with Race Reporting | Self-Report: Youth Behavior Risk Surveillance; Monitoring the Future | Self-Report: CASI; RCADS | Anxiety sensitivity symptoms associated with more negative cannabis effects of cannabis (p <.05); Higher GAD symptoms initially report more positive cannabis effects, but have a slower increase in these effects across time (β = −0.06; p = 0.02) |  |
| Rusby et al., 2019 | United States | High School | 14.4 (Spring of 8th grade-10th grade) | 466 (52.8%) | 44% White, Non-Hispanic; 38% Hispanic; 18% Other or multiple races | Combined with Race Reporting | Self-Report: Oregon Healthy Teens Survey | Self-Reported Mood (EMA) | Recent cannabis use associated with significantly greater anxious mood lability (p < .001) |  |
| Schuster et al., 2019 | United States | Urban/ City Environment | 21.79 (18-25) | 76 (44.7%) | 63.2% White;  15.8% Black;  11.8% More than one race; 9.2% Other | NR | Self-Report: Cannabis Use Disorder Identification Test-Revised; Modified Timeline Follow-back; Marijuana Effect Expectancy Questionnaire.  Biometric Data: Urine Toxicology Screen | Self-Report: Mood and Anxiety Symptom Questionnaire (MASQ) | Current anxious symptoms (p= 0.01) and current anxious arousal (p = 0.0002) associated with greater cannabis use dependency in young adults who use cannabis at least weekly |  |
| Stapinski et al., 2016 | Chile | Urban/ City Environment, High School | 14.5 (12-18) | 2,508 (44.5%) | NR, Schools in Santiago, Chile | NR | Self-Reported Use | Self-Report: RCADS | Cannabis use frequency at 18 month follow-up associated with baseline generalized anxiety (p = 0.002) |  |
| Thompson et al., 2018 | Canada | Urban/ City Environment | 15.5 at T1 (12-22; 18-29 ranges across timepoints) | 662 (50.3%) | NR | NR | T1: Self-Reported Use T6: Clinical Interview: MINI | Self-Report: Brief Child & Family Phone Interview (BCFPI) | Chronic users reported higher levels of anxiety symptoms compared with abstainers and decreasers (p < .05); for young adults, chronic users reported more anxiety symptoms than all other classes except occasional users. |  |
| Villarosa-Hurlocker et al., 2019 | United States | University/ College | 20.24 (NR) | 2,034 (69.1%) | NR | 67.95% White, Non-Hispanic; 15.88% Hispanic/Latino Ethnicity | Self-Report: 21-item Brief Marijuana Consequences Questionnaire | Self-Report: SIAS; Brief Fear of Negative Evaluation | When controlling for all other predictors, social anxiety associated with more cannabis-related problems |  |
| Wolitzky-Taylor et al., 2016 | Unites States | High School | 14.1 (NR) | 3,002 (54.1%) | 47.4% Hispanic; 6.7% Multiracial; 16.6% Asian;  16.1% Caucasian;  4.9% African American; 4.1% Native Hawaiian or Pacific Islander;  1% American Indian or Alaska Native | Combined with Race Reporting | Self-Report: CAST | Self-Report: RCADS | Negative urgency mediated associations between cannabis use and GAD (b = .06, p < .001), panic disorder (b = .05, p < .001), and social phobia (b = .04, p < .001). |  |
| **Unclear or No Association** | | | | | | | | | | |
| Buckner et al., 2016 | United States | University/ College | 20.2 (18-29) | 276 (79.7%) | 76.1% Non-Hispanic White;  12.0% Non-Hispanic African American;  4.7% Multiracial; 2.5% Asian or Asian American; 2.2% Hispanic White;  1.1% Other;  1.0% American Indian or Alaska Native;  .4% Hispanic African American | Combined with Race Reporting | Self-Report: MUF; Investigator-developed questionnaire adapted from Gonzalez & Skewes | Self-Report: SIAS | Cannabis problems more likely related to solitary use than social anxiety experiences |  |
| Butler et al., 2019 | Canada | High School | NR | 6550 (51.7%) | 71.5% "Non-minority ethnicity" | 71.5% "Non-minority ethnicity" | Self-Reported Use | Self-Report: GAD-7 | Although anxiety (p=0.001) was found to be associated with cannabis use frequency in model 4, anxiety was not associated with the frequency of cannabis use after including flourishing in model 5 and 6. |  |
| Cerdá et al., 2016 | United States | High School | NR (13-19) | 503 (0%) | 56% Black; 41% White; 3% Asian, Mexican, Mixed Race | Partially combined with Race Reporting/NR | Self-Report: SRA; 16-item Substance Use Scale based on the National Youth Survey | Self-Report: CBCL | Higher anxiety and affective problems not associated with use |  |
| Cloutier et al., 2016 | United States | Community Environment | 16.2 (12-17) | 56 (41.1%) | 83.9% Caucasian; 5.4% African American;  7.1% multiracial;  3.6% “Other” | 9.1% Hispanic/Latino | Self-Report: AADIS; TMMQ | Self-Report: RCADS (social anxiety subscale); Youth Self-Report – Anxiety problems subscale | No association with anxiety across measures of use frequency |  |
| Colder et al., 2019 | United States | Community Environment | 12 (11-12) | 387 (55%) | 83.1% non-Hispanic Caucasian;  9.1% African American | Partially combined with Race Reporting/NR | Self-Report: Use Frequency items; MMQ; MACQ | Self-Report: SIAS | Elevations in social anxiety were not associated with coping motives, or with cannabis use or problems |  |
| Ecker et al., 2014 | United States | University/ College | 20.28 (18-NR) | 158 (75.3%) | 77.2% Caucasian;  8.9% African American; 8.2% Mixed; 5.1% Asian; .6% Other | 8.2% Hispanic/Latino | Self-Report: Daily Drug-Taking Questionnaire; Marijuana Problem Scale | Self-Report: Social Phobia Scale | Non-significant correlation between cannabis use frequency and social anxiety |  |
| Elkington et al., 2016 | United States | Urban/ City Environment, Community Environment | 12.58 (9-16) | 340 (51.2%) | PHIV+  57.8% Black; 30.1% Hispanic; 12.1% Other PHIV-  49.3% Black; 32.1% Hispanic; 18.6% Other | Combined with Race Reporting | Clinical Interview: DISC-IV | Clinical Interview: DISC-IV | Frequency of use not associated with having an anxiety disorder at baseline, follow-up points |  |
| Gage et al., 2015 | United Kingdom | Rural Environment | 16, 18 (Prospective assessments) | 4,561 (NR) | NR | NR | Self-Reported Use | Clinical Interview: CIS-R | No significant relationship |  |
| Gillen et al., 2016 | United States | Residential military-style program in the southeastern United States for youth who have dropped out of high school | 16.74 (16-19) | 185 (0%) | 54.1% White; 24.3% Black; 18.4% Did not report;  1.6% Another ethnic group;  1.1% Hispanic;  .5% Asian | Combined with Race Reporting | Self-Report: CRAFFT; MMQ | Self-Report: Personality Inventory for Youth (Fear and Worry Subscale) | No significant relationship |  |
| Grunberg et al., 2015 | United States | University/ College | 18.30-18.38 (18-21) | 375 (46.9%) | 73% White; 18.7% Multi-Racial; 3.6% Asian; 3.3% Hispanic; .6% East Indian; .3% Black; .3% Middle Eastern .3% Pacific Islander | Combined with Race Reporting | Self-Report: TLFB | Self-Report: ASEBA | T1 cannabis use not correlated with T1 or T2 anxiety |  |
| Khoddam et al., 2016 | United States | High School | 14.1 (NR) | 3,383 (53%) | 45.9% Hispanic or Latino; 15.8% Asian; 15.3% White; 5.9% Multiracial; 5.6% Other; 4.9% Black/African American; 3.3% Native Hawaiian/Pacific Islander; 0.9% American Indian/Alaska Native | Combined with Race Reporting | Self-Reported Use | Self-Report: RCADS | Anxiety symptoms not associated with use over and above conduct problems |  |
| Ninnemann et al., 2017 | United States | High School | 16.09 (NR) | 964 (56%) | 31% African American; 29% White; 28% Hispanic; 12% Other | Combined with Race Reporting | Self-Report: MTF | Self-Report: Screen for Child Anxiety-Related Emotional Disorders | No significant relationship |  |
| Osuch et al., 2013 | Canada | Urban/ City Environment, Community Environment | 19 (16-26) | 429 (63.2%) | NR | NR | Self-Report: NIDA Modified ASSIST | Self-Report: STAI | No significant relationship |  |
| Phillips et al., 2018 | United States | University/ College | 20.32 (18-25) | 300 (60%) | 69% Caucasian (Non-Latino/Hispanic); 15% Latino/Hispanic; 6% African American;  6% Multi-racial; 2% Asian; 2% Pacific Islander/Hawaiian | Combined with Race Reporting | Self-Report: Marijuana Use Measure; Rutgers Marijuana Problem Index Biometric Data: Urine Toxicology Screen | Self-Report: BAI; SIAS | No significant relationship |  |
| Rahm-Knigge et al., 2019 | United States | University/ College | 18.92 (NR) | 1,005 (67.6%) | 83.75 White; 5.1% Multi-racial; 4.0% Asian; 3.0% Do not wish to respond; 2.2% Black or African American; .9% American Indian or Alaskan Native;  0.9% Native Hawaiian or Other Pacific Islander | 81% Not Hispanic or Latino; 15.6% Hispanic or Latino; 2.5% Do not wish to respond; .9% Missing | Self-Report: Risky Behavior Inventory | Self-Report: SAIS | No direct association, but certain profiles (Low Social Interaction Anxiety High Urgency) may be more likely to endorse use |  |
| Walters et al., 2018 | United States | University/ College | 20.37 (18-25) | 891 (69.6%) | 69% White; 15% Black/African American; 8% Hispanic/Latino;  6% Other; 2% Asian/Pacific Islander | Combined with Race Reporting | Self-Report: CORE Alcohol and Drug Survey – Short form | Self-Report: PAI | No significant relationship |  |
| Wright et al., 2016 | United States | University/ College | 21.2 (18-25) | 84 (46.4%) | Control: 60% Caucasian;  Cannabis Users: 67% Caucasian | NR | Self-Report: TLFB; Clinical Interview: Semi-structured interview | Self-Report: STAI | Overall, no association; Female cannabis users more likely to experience anxiety symptoms (p = .04) |  |

*Note.* Race and ethnicity categories are reported as described in the articles. AADIS = Adolescent Alcohol and Drug Involvement Scale; AS = Original 16-item Anxiety Sensitivity Index; ASA-27 = Adult Separation Anxiety Questionnaire; ASEBA = Achenbach System of Empirically Based Assessment Self-report; B-MACQ = Brief Marijuana Consequences Questionnaire; BAI = Beck Anxiety Inventory; BCFPI = Brief Child & Family Phone Interview; BFNE = Brief Fear of Negative Evaluation; BPRS = Brief Psychiatric Rating Scale; BSI = Brief Symptom Inventory; CAPA = Child and Adolescent Psychiatric Assessment; CASI = Childhood Anxiety Sensitivity Index; CAST = Cannabis Abuse Screening Test; CBCL = Child Behavior Checklist; CIS-R = Clinical Interview Schedule-Revised; CPQ-A = Adolescent Cannabis Problems Questionnaire – Short Form; CUDIT-R = Cannabis Use Disorder Identification Test-Revised; CUPIT = Cannabis Use Problems Identification Test; DASS-21 = Depression, Anxiety, Stress Scale; DISC-IV = Diagnostic Interview Schedule for Children-IV; EMA = ecological momentary assessment; ESPAD = European School Survey Project on Alcohol and Other Drugs questionnaire; GAD-7 = Generalized Anxiety Disorder-7; HSCL = Hopkins Symptom Checklist; LSAS-CA-SR = Liebowitz Social Anxiety Scale for Children and Adolescents-Self-Reported version; MACQ = Marijuana Adult Consequences Questionnaire; MASQ = Mood and Anxiety Symptom Questionnaire; MEEQ = Marijuana Effect Expectancies Questionnaire; MINI = Mini-International Neuropsychiatric Interview; MMQ = Marijuana Motives Questionnaire; MPI = Rutgers Marijuana Problem Index; MPS = Marijuana Problems Scale; MTF = Monitoring the Future survey; MUF = Marijuana Use Form; NIDA Modified ASSIST = National Institute of Drug Abuse modified version of the WHO Alcohol, Smoking and Substance Involvement Screening Test, version 3.0; NR = not reported; OHT = Oregon Healthy Teens Survey; PAI = Personality Assessment Inventory; RCADS = The Revised Children’s Anxiety and Depression Scale; SAS = Zung Self-Rating Anxiety Scale; SIAS = The Social Interaction Anxiety Scale; SRA = Self-Reported Antisocial Behavior Scale; SCL-90R = Symptom Checklist-90-R; STAI = State-Trait Anxiety Inventory; STAI-CH = State-Trait Anxiety Inventory for Children-Trait Subscale; SURPS = Substance Use Risk Profile Scale; TLFB = Timeline Follow-Back; TMMQ = Teen Marijuana Motives Questionnaire; YAPA = Young Adult Psychiatric Assessment; YRBSS = Youth Behavior Risk Surveillance
